# Embryonic Exposure to TPhP Elicits Osteotoxicity via Metabolic Disruption in *Oryzias latipes*

**DOI:** 10.3390/toxics13080654

**Published:** 2025-07-31

**Authors:** Melissa C. Gronske, Jamie K. Cochran, Jessika D. Foland, Dereje Jima, David B. Buchwalter, Heather M. Stapleton, Seth W. Kullman

**Affiliations:** 1 Comparative Biomedical Sciences, College of Veterinary Medicine, North Carolina State University, Raleigh, NC 27695, USA; 2 Toxicology Program, North Carolina State University, Raleigh, NC 27695, USA; 3 Department of Biological Sciences, North Carolina State University, Raleigh, NC 27695, USA; 4 Center for Human Health and the Environment, North Carolina State University, Raleigh, NC 27695, USA; 5 Nicholas School of the Environment, Duke University, Durham, NC 27710, USA

**Keywords:** triphenyl phosphate, medaka, metabolic disruption, skeletal toxicity

## Abstract

Triphenyl phosphate (TPhP) is a widely used organophosphate flame retardant and plasticizer, raising concerns over its health impacts. This study examined the effects of embryonic TPhP exposure on axial skeletal development and metabolism in medaka (*Oryzias latipes*), a vertebrate fish model relevant to human bone biology. Medaka embryos were exposed to 1 µM TPhP and assessed through early larval stages. TPhP impaired vertebral ossification, causing shortened centra and reduced cartilage in the caudal complex, alongside disrupted distribution of osteoblast-lineage cells. Key osteogenic genes were significantly downregulated at 14 days post fertilization, and transcriptomic analysis revealed altered mitochondrial pathways linked to skeletal disorders. Functionally, TPhP-exposed larvae showed reduced caudal fin regeneration and decreased metabolic rate and oxygen consumption, consistent with mitochondrial dysfunction. These findings indicate that TPhP disrupts bone development and metabolism by affecting osteoblast differentiation and mitochondrial regulation, highlighting the value of small fish models for studying environmental toxicants and bone metabolic disease risk.

## 1. Introduction

Bone disorders encompass a wide range of conditions that impact both early development and later stages of life, presenting significant health and socioeconomic challenges worldwide. Congenital skeletal disorders, such as skeletal dysplasias, are typically diagnosed in infancy or early childhood and result from genetic mutations that impair the normal formation and growth of bone and cartilage. These conditions are diverse, with over 400 distinct types recognized, and they occur at a rate of approximately 1 in every 5000 live births [1,2]. Advances in molecular genetics have identified numerous pathogenic variants affecting key regulators of bone and cartilage development, shedding light on the etiology and classification of these disorders [3]. In contrast, age-related skeletal diseases, including osteopenia, osteoporosis, osteoarthritis (OA), and rheumatoid arthritis (RA), predominantly affect older adults. These disorders are often characterized by bone and cartilage degradation, impaired structural integrity, and increased susceptibility to fractures or joint dysfunction [4,5]. Osteoporosis alone affects approximately 200 million people worldwide and is a leading cause of fragility fractures in aging populations [6]. Together, developmental and degenerative skeletal disorders impose a considerable burden on public health, highlighting the need for continued research into their underlying mechanisms and therapeutic interventions.

Exposure to exogenous factors is increasingly recognized as a key contributor to skeletal diseases, including skeletal dysplasia, degenerative bone conditions, and impaired fracture repair. Cigarette smoke, alcohol use, poor nutrition (obesity, vitamin D deficiency, etc.), and environmental toxicants are established risk factors for poor skeletal outcomes [7]. Heavy metals, pesticides, endocrine disruptors, per- and polyfluoroalkyl substances (PFAS), and dioxin-like compounds are demonstrated to disrupt osteogenesis, impact long-term bone health, and contribute to degenerative diseases by affecting inflammation, cytokine production, bone remodeling, and mesenchymal stem cell (MSC) recruitment and differentiation [8]. The skeletal system is additionally vulnerable due to its ability to sequester toxicants like heavy metals and the precision required by its highly conserved developmental processes [9,10,11]. This prolonged contact with harmful substances allows toxicants to interfere with critical cellular functions by inappropriately activating signaling pathways or inhibiting the activity, synthesis, or secretion of essential proteins [11,12,13,14].

Flame retardants are a broad group of chemicals incorporated into materials such as plastics, textiles, electronics, and building products to reduce flammability and help meet fire safety standards [15,16]. Historically, polybrominated diphenyl ethers (PBDEs) were among the most widely used flame retardants, applied extensively across consumer goods and construction materials [17]. Following regulatory actions and voluntary phase-outs, alternative flame retardants such as organophosphate esters (OPFRs) have increasingly replaced PBDEs in many applications [18,19]. These compounds are now commonly detected in indoor environments and global ecosystems, reflecting their widespread use and release [18]. Among these, TPhP is commonly detected in the environment, with concentrations typically ranging from ng/L to low µg/L in surface waters and up to 51 µg/g in indoor dust, demonstrating its pervasive global presence [20,21,22]. Despite its moderate lipophilicity (log K_ow ~4.6), TPhP exhibits variable bioaccumulation in aquatic organisms depending on the species and exposure conditions [23,24]. Human biomonitoring studies consistently detect TPhP exposure with urinary concentrations typically in the low ng/mL range, and TPhP or its metabolites have also been found in human hair, nails, and breast milk, suggesting systemic distribution and potential maternal transfer [25,26,27,28]. Together, these findings emphasize that TPhP is a ubiquitous environmental contaminant with demonstrated potential for bioaccumulation and biological effects across species, underlining the need for continued research into its ecological and human health impacts.

Growing evidence increasingly implicates triphenyl phosphate (TPhP) and other organophosphate flame retardants (OPFRs) in a range of adverse health effects, including neurotoxicity, developmental toxicity, immunotoxicity, endocrine disruption, and carcinogenicity [19,29,30,31,32,33,34]. Notably, as an endocrine-disrupting compound, TPhP has been shown to interfere with skeletal development, where embryonic exposures to TPhP in mouse embryonic limb bud cultures disrupts endochondral ossification at concentrations as low as 1–3 µM, indicating its potential to impair bone formation during critical developmental windows [35]. Consistent with these findings, aquatic models demonstrate significant osteotoxic effects in marine medaka (*Oryzias melastigma*) and zebrafish (*Danio rerio*) larva, resulting in attenuation of larval body length and formation of tail malformations [36,37]. Furthermore, chronic two-generational exposure to TPhP in marine medaka results in persistent developmental defects such as spinal curvature and pectoral fin abnormalities, highlighting the chemical’s potential for transgenerational developmental toxicity and long-term health impacts [36]. Together, these studies underscore the multifaceted toxicity of TPhP and its potential to disrupt critical biological processes across species and developmental stages.

This study explores the skeletal toxicity of TPhP, a widely used organophosphate flame retardant. Given the tightly regulated nature of skeletal development and its continual remodeling, the skeleton represents a sensitive target for toxicological disruption. Using embryos and larvae of medaka, we examined the developmental impact of TPhP exposure on skeletal formation and assessed fin ray regeneration as a measure of bone remodeling capacity. We evaluated skeletal morphology, larval metabolic rate, and the expression of genes and pathways involved in axial skeletal development. Our findings show that embryonic exposure to TPhP leads to impaired skeletal mineralization, vertebral and caudal fin malformations, and reduced metabolic function in medaka larvae.

## 2. Materials and Methods

***Medaka housing and care:*** All medaka (*Oryzias latipes*) in this study were cared for in accordance with standard protocols approved by the North Carolina State University Institutional Animal Care and Use Committee (#22–378). Adult medaka were housed at appropriate densities in 10 L and 30 L tanks in an enclosed, recirculating aquaculture system under a 14:10 h light/dark cycle. Conditions were monitored daily, maintaining temperature at 26 ± 2 °C and pH at 7.2 ± 0.2. The inbred orange-red Hd-dR (OR) and previously described transgenic strains [38] were used for the following experiments. All fish were monitored daily to ensure there was no change in health and prevent pain and suffering. TPhP exposure did not result in any physical harm to the animals, and no fish had to be sacrificed due to a decline in health and or increased stress from exposures. Additionally, none of the assessed endpoints induced any harm to any fish. Upon completion of each experiment, fish were euthanized using ice water at indicated time points for morphological assessment or RNA collection.

***Triphenyl phosphate exposure:*** OR embryos were collected from breeding tanks, cleaned, and staged by previously described methods [39]. Fertilized embryos were transferred to glass crystallizing dishes containing 10 mL of 1X embryo rearing medium (ERM), as previously described [38], at a density of 20–30 embryos per dish using four replicates dishes per treatment. Exposure to 0.1% dimethyl sulfoxide (DMSO) or 1.0 µM of TPhP (Sigma Aldrich) in DMSO began during early development at 4–6 h post fertilization (hpf) (blastula stage). The use of 1.0 µM TPhP was based upon previous studies in aquatic models demonstrating non-lethal, sub-chronic developmental effects on embryonic development following aqueous exposures to TPhP [36,37]. Embryos were cultured at 26–28 °C with static media with compound renewal (~80%) every 2–3 days until termination of the experiment at 14–20 days post fertilization (dpf). Free-swimming hatched larvae were fed 1–2 mg of ground Otohime B1 larval diet (Pentair) daily until termination.

***Whole-mount histological staining for bone and cartilage:*** Larvae were euthanized and stained at 20 dpf with an acid-free double stain solution (Alizarin red and Alcian blue), as previously described by Walker and Kimmel [40]. Briefly, larvae were anesthetized in ice water for 10–20 min and then immersed in a fixative solution containing 4% paraformaldehyde (PFA) in 1X phosphate-buffered saline (PBS), pH−7.0 (Thermo Fisher Scientific, Waltham, MA, USA) and gently agitated overnight at room temperature. Specimens selected for dual bone and cartilage staining were washed four times for 10 min each with 1% PBS before being washed and dehydrated with 50% ethanol, with rocking, at room temperature for 10 min. An acid-free double stain was made in two parts mixed just before staining. Part A contains 0.02% Alcian blue, 60 mM MgCl_2_, and 70% ethanol, while part B contains 0.5% Alizarin red S powder dissolved in water. Just before staining, the two parts were mixed in a ratio of 10 µL of part B to 1 mL of part A. Once the 50% ethanol was removed, the double stain was added to the larvae and rocked overnight at room temperature. Stained larvae were bleached in a 3% H_2_O_2_ and 1% KOH solution for 20 min at room temperature. Larvae were then cleared with a 20% glycerol and 0.25% KOH solution overnight before replacement with a 50% glycerol and 0.25% KOH solution. Samples were subjected to a graded series of 50%, 75%, and 90% glycerol in 0.1% KOH and stored in 100% glycerol at room temperature. Representative larvae from each treatment group (*n* = 8–10) were imaged under light microscopy using a Nikon SMZ1500.

***In vivo staining for bone mineralization and confocal microscopy*:** At 14 dpf, live medaka larvae treated with either DMSO or TPhP were stained and imaged to evaluate the localization of key cellular populations involved in osteoblast and chondrocyte development relative to mineralized bone. Specific transgenic reporter lines were used to label distinct cell types: mesenchymal progenitors including *tg*(*twist1:EGFP*), early osteoblasts *tg(col10a1:nlGFP)*, mature osteoblasts *tg*(*osx*/*sp7:mCherry*), and early chondrocytes *tg(col2a:EGFP).* Approximately 12 h before imaging, larvae were counterstained with 0.1% alizarin complexone (ALC; Sigma Aldrich, St. Louis, MO, USA) in embryo rearing medium (ERM) for 1 h at 26 °C or with 0.2% calcein (Sigma Aldrich) in ERM for 10 min at 26 °C. Following staining, larvae were washed twice in 1X ERM and incubated overnight in fresh ERM to allow for equilibration of the fluorescent dyes. Representative larvae from each treatment group (*n* = 8–10) were anesthetized in 0.03% tricaine (*w*/*v*) in ERM and mounted laterally in 35 mm glass-bottom dishes (MatTek Corporation, Ashland, MA, USA) to facilitate imaging of the axial vertebrae (vertebrae 17–19) and the caudal skeletal complex. In vivo imaging was performed using a Zeiss LSM 880 laser scanning confocal microscope (Carl Zeiss Microscopy GmbH, Jena, Germany) equipped with ZEN 2012 version 2.3 SP1 image acquisition software.

***Morphological assessment:*** Maximum intensity projections of confocal z-stack images from *tg(twist1:EGFP)*/ALC, *tg(osx/sp7:mCherry)*/Calcein, *tg(col2a:EGFP)*/ALC, and *tg(col10a1:nlGFP)*/ALC medaka larvae were generated using ZEN 2012 Imaging Software to produce two-dimensional representations of the axial vertebrae and caudal complex for morphological assessment. Gross evaluation included examination of the caudal skeletal elements and confirmation of mineralized centra and associated neural and hemal arches. For each treatment group, images from whole-mount stained individuals were analyzed using ImageJ V1.54 g (NIH) [41] to quantify the following morphometric features: area of mineralized centra (mm^2^), area of the intervertebral ligament (mm^2^), area of hypurals 1 and 2 (HP1, HP2) (mm^2^), area of the parahypural (PH) (mm^2^), area of the hemal arch of preural centrum 2 (HPU2) (mm^2^), and the lengths of PH and HPU2 (mm). For each structural feature, three independent measurements per individual (*n* = 8–10) per treatment group were averaged, and the data were then pooled to calculate means for vertebrae 17–19 and caudal fin components. Morphometric values from DMSO-treated larvae were normalized to 1, and values from TPhP-treated larvae were expressed as a proportion relative to DMSO controls.

***RNA isolation****:* At 14 dpf, larval medaka were euthanized in ice-cold embryo rearing medium (ERM) and pooled in microcentrifuge tubes to obtain 15–20 individuals per RNA sample. Residual water was removed, and Leibovitz’s L−15 medium (Corning, Mediatech, Inc., Manassas, VA, USA) supplemented with 10% fetal bovine serum (FBS; Atlanta Biologicals) was added. To isolate axial skeletal tissue, hatched larvae were passed multiple times through a 20–22-gauge hypodermic needle attached to a 3 mL syringe (Becton, Dickinson and Company, Franklin Lakes, NJ, USA). The resulting tissue suspension was filtered through 105 μm nylon mesh (Component Supply Co., Fort Meade, FL, USA) and transferred to fresh L−15/10% FBS medium. Under a dissecting microscope (Nikon SMZ1500, Nikon Instruments, Inc., Melville, NY, USA), axial tissue was carefully examined and trimmed to remove any remaining craniofacial or abdominal tissue. Cleaned samples were immediately flash-frozen in liquid nitrogen. Tissues were homogenized in TRI Reagent^®^ (Ambion^®^, Life Technologies, Carlsbad, CA, USA) using a handheld BioVortexer (Thomas Scientific, Swedesboro, NJ, USA), and total RNA was extracted following the manufacturer’s protocol. RNA concentration was determined using the Qubit^®^ RNA HS Assay Kit (Invitrogen, Carlsbad, CA, USA) with the Qubit^®^ 3.0 Fluorometer.

***qPCR****:* Complementary DNA (cDNA) was synthesized from 1 µg of total RNA using the High-Capacity cDNA Reverse Transcription Kit (Applied Biosystems, Foster City, CA, USA). Each 20 µL reaction included random primers, MultiScribe™ Reverse Transcriptase, RNase inhibitor, dNTP mix, and 10X reverse-transcription buffer. Gene-specific primers were designed using Primer3 and synthesized by Integrated DNA Technologies (IDT) (Supplemental Table S1). Relative gene expression was quantified via real-time PCR using cDNA from DMSO- and TPhP-treated samples (*n* = 3–4 technical replicates of 3–4 biological replicates of 15–20 pooled larvae), with each reaction performed in triplicate on clear 96-well PCR plates (Olympus Plastics, Genesee, San Diego, CA, USA) using the QuantStudio™ 3 Real-Time PCR System. Each 20 µL qPCR reaction consisted of 10 µL of SYBR Green Master Mix (Life Technologies), 0.4 µL of 10 µM forward primer, 0.4 µL of 10 µM reverse primer, 1.6 µL of cDNA, and 7.6 µL of UltraPure™ distilled H_2_O (Life Technologies). PCR cycling conditions were as follows: (1) 50 °C for 2 min, (2) 95 °C for 10 min, (3) 40 cycles of 95 °C for 15 s and 60 °C for 60 s, and (4) a dissociation stage consisting of 95 °C for 15 s, 60 °C for 60 s, 95 °C for 15 s, and 60 °C for 15 s to generate melt curves and confirm primer specificity. Cycle threshold (Ct) values were determined using QuantStudio™ Design & Analysis Software v1.5.2 (Applied Biosystems), and relative gene expression was calculated using the ΔΔCt method [42], with normalization to the reference gene *RPL13α*. All primer pairs were assessed for optimal efficiency between 90–110%, and negatives (no template controls) were conducted for each experimental cDNA/primer pair.

***RNA-Seq****:* Illumina RNA library preparation and sequencing were performed at the NC State Genomic Sciences Laboratory (Raleigh, NC, USA). Total RNA was extracted from DMSO- and TPhP-treated embryos/larvae (*n*= 3–4 technical replicates of 3–4 biological replicates of 15–20 pooled larvae) and assessed for quality, concentration, and integrity using RIN ≥ 9 and an Agilent 2100 Bioanalyzer with an RNA 6000 Nano Chip (Agilent Technologies, Cary, NC, USA). From 1 µg of total RNA, messenger RNA (mRNA) was purified using oligo-dT magnetic beads from the NEBNext Poly(A) mRNA Magnetic Isolation Module (New England Biolabs, Ipswich, MA, USA). Complementary DNA (cDNA) libraries were generated using the NEBNext Ultra Directional RNA Library Prep Kit and NEBNext Multiplex Oligos for Illumina, following the manufacturer’s instructions. In brief, purified mRNA was chemically fragmented and reverse-transcribed into first-strand cDNA using random primers. Second-strand synthesis incorporated dUTPs to retain strand specificity. The resulting double-stranded cDNA was purified, end-repaired, and adenylated prior to adapter ligation. Size selection was performed using AMPure XP beads (Beckman Coulter, Brea, CA, USA) to isolate library fragments between 400 and 550 bp (including adapters). Libraries were then PCR-amplified to enrich fragments and incorporate unique index barcodes. Final libraries were purified and assessed for size distribution and concentration using an Agilent 2200 TapeStation with a High-Sensitivity DNA chip and a Qubit fluorometer (Thermo Fisher, Raleigh, NC, USA). Indexed libraries were pooled in equimolar amounts and sequenced on an Illumina HiSeq 2500 platform using a 125 bp single-end sequencing kit. Base calling was conducted with Real-Time Analysis (RTA) (V 2.4.11) software to produce raw BCL files, which were subsequently demultiplexed into FASTQ files for downstream analysis.

Bioinformatics processing and data interpretation were carried out in consultation with the Bioinformatics Core at the NCSU Center for Human Health and the Environment. The quality of sequenced data was assessed using FastQC, and poor-quality bases were trimmed from the 5′-end. The remaining good-quality reads were aligned to the medaka reference genome (ASM223467V1) using STAR aligner [43]. For each replicate, per-gene counts of uniquely mapped reads were calculated using htseq-count script from the HTSeq python package (V 2.0.9). We imported the count matrix to the R statistical computing environment (V 4.5.1) for further analysis. Gene count data were normalized for sequencing depth and distortion, and dispersion was estimated using DESeq2 Bioconductor package (V 1.40.2) in the R statistical computing environment [44]. We fitted a linear model using the treatment levels, and differentially expressed genes were identified after applying multiple testing corrections using the Benjamini–Hochberg procedure (padj < 0.05) [45]. Ingenuity Pathway Analysis (IPA, Qiagen, Valencia, CA, USA) version 01–10(01–10) provided predictive models of pathway modulation based on transcript abundance (control vs. TPhP treatment) [46]. Gene set enrichment analysis (GSEA (version 4.0.3)) software was used to show statistically significant differences between control and TPhP treatments [47]. Briefly, we pre-ranked all genes that were measured using RNA-Seq using fold-change * -log10(p value) and sorted in descending order. Enrichment analyses were conducted against curated C2 all-version 7.1 symbols. In the GSEA application, we modified the following parameters (enrichment statistics: classic, exclude large sets: 5000, exclude smaller sets: 15). We then assessed the top 20 differentially enriched gene sets and chose to present the most relevant gene set based on our described phenotypes.

***Fin Clip Regeneration:*** Larvae at 14 dpf (*n* = 35–48 larvae per treatment) were briefly anesthetized in ice cold 1X ERM. Once anesthetized, larvae were placed in a dissecting dish under a microscope with a thin layer of 1X ERM. Fin clips were then performed with a dissection blade. Larvae were returned to either 1X ERM containing either 1.0 µM TPhP or DMSO and incubated at 26 °C for 4 days under appropriate care and feeding. At 19 dpf, larvae were again briefly anesthetized, and fin growth was imaged under the dissection scope. Images were processed using ImageJ to measure the “stump width” (STU) and “regeneration area” (REG) in pixels [48]. The individual REGs were normalized to individual STUs to calculate the REG/STU ratio for comparison between treatment groups.

***Microrespirometry:*** A closed 24-well glass microplate system equipped with 24 optical sensor spots (one per well) (Loligo Systems, Viborg, Denmark) was used for all microrespirometry experiments. Medaka larvae (*n* = 5–8 larvae per treatment) were pretreated from early development at 4–6 hpf to 13 dpf with either TPhP (1.0 uM) or DMSO (0.1%), depurated for 24 h in clean 1X ERM, and assessed for oxygen consumption at 14 dpf. Oxygen consumption was measured by a 24-channel SensorDish reader (SDR; PreSens Precision Sensing, Regensburng, Germany). The wells were filled with 1X ERM and cleared of air bubbles using forceps, and then, individuals were added (one individual per each 500 uL well). The microplate was then sealed with Whatman™ UniSeal™ adhesive clear seal film and then submerged in a water bath. Temperature was maintained at 27.1 °C using a Cole-Parmer^®^ programmable Polystat^®^ temperature controller, which constantly circulated water through the water bath. Individuals were monitored for 4.5 h, as they depleted the oxygen within the well. MicroResp software (v1.0.4) (Loligo Systems, Viborg, Denmark) logged dissolved oxygen (DO) (mg/L) every 15 s. Individuals were subsequently dried overnight at 60 °C, and dry weight was obtained to the nearest 0.001 mg using a Sartorius CPA2P microbalance. DO measurements (as obtained from MicroResp software) were corrected for weight and respiratory volume before being plotted against time (in hours). The slope of the resulting line was taken as the SMR (in μg O_2_ g^−1^ h^−1^). The critical oxygen partial pressure (P_crit_) was determined as the point where the linear rate of oxygen changed significantly at lower oxygen partial pressures. Blank wells insured that microbial activity in ERM was negligible and did not positively bias measurements of oxygen consumption.

***Statistical Analysis:*** Results for the assays described above are presented as the mean ± SD. For the caudal complex morphological assessment, *n* = 8–10 replicates. For the morphological assessment of fin growth, *n* = 35–48 replicates. For the metabolic function test, *n* = 5–8 replicates. For qPCR analysis, *n* = 3–4 technical replicates of 3–4 biological replicates of 15–20 pooled larvae. Pairwise comparisons between DMSO and TPhP were conducted using an unpaired, two-tailed Student’s *t*-test. For all statistical analyses, a significance threshold of α = 0.05 was applied, with *p*-values below this threshold considered statistically significant.

## 3. Results

**Figure 1:**
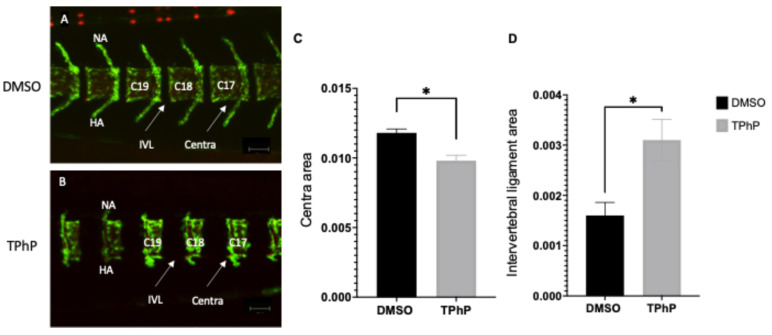
Morphological assessment of axial structures (V16–21) of tg(Col10a:egfp) medaka larvae at 14 dpf. Representative individual from DMSO (**A**) and 1.0 µM TPhP-exposed (**B**) groups are shown. Individuals were counterstained with ALC (red) to identify mineralized bone matrix within the context of osteoblasts and col10a (green) expressing osteoblast precursors. Images were captured using the Zeiss LSM 880 confocal system, 10× magnification (scale bars = 100 µm). Morphometric assessment of centra (V17–19) area (**C**) and intervertebral ligament (IVL) area (**D**). Values represent the mean of 8–10 independent measurements taken from vertebrae 17–19. Statistical differences between groups were assessed using Student’s *t*-test. Error bars represent SD, and asterisks denote statistical significance at (*p* ≤ 0.05). C17–19, centra 17–19 (rostral-caudal); HA, hemal arch; NA, neural arch; IVL, intervertebral ligament.

**Figure 2:**
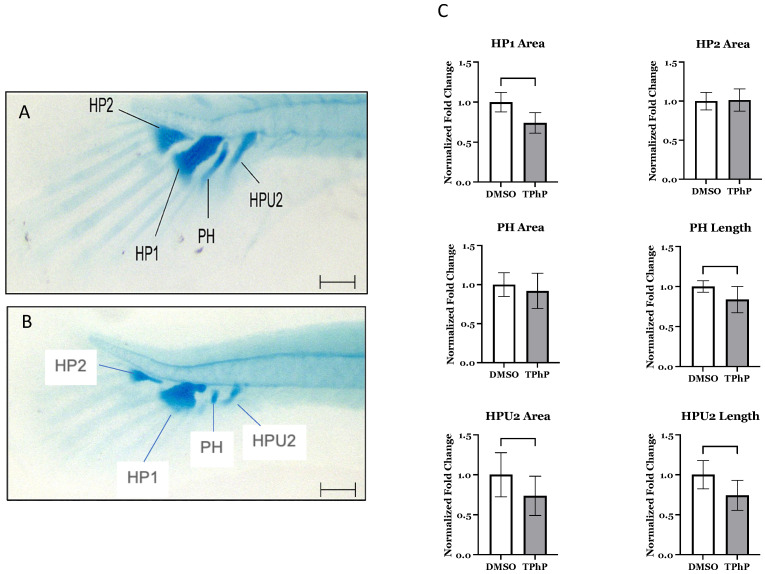
Morphological assessment of caudal complex structures of whole-mount Alcian blue staining for cartilage in medaka larvae at 20 dpf. Representative individuals from DMSO- (**A**) and 1.0 µM TPhP-exposed (**B**) groups are shown, 3.2× magnification. Total area of hypural structures are shown in (**C**), where DMSO values are normalized to 1. Values represent the mean from 8–10 separate measurements. Error bars represent SD. Scale bars in (**A**,**B**) represent 100 µm.

**Figure 3:**
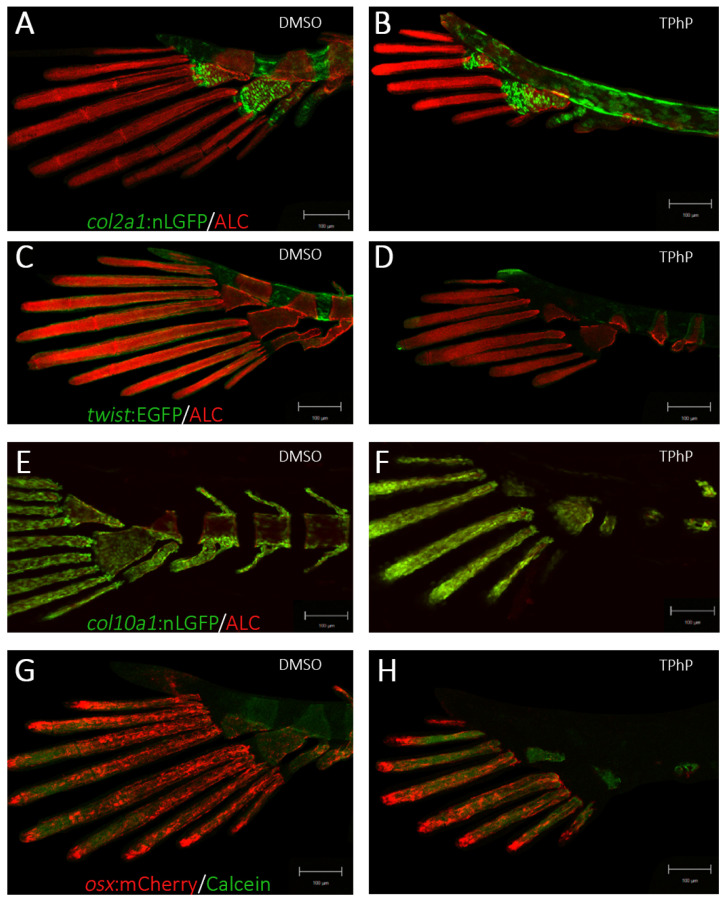
Representative confocal images of 14 dpf medaka exposed to DMSO or TPhP in the following transgenic lines: tg(col2a1:nlGFP) (**A**,**B**), tg(twist:EGFP) (**C**,**D**), tg(col10a1:nlGFP) (**E**,**F**), and tg(osx/sp7:mCherry) (**G**,**H**). Specimens were counterstained with Alizarin Complexone (ALC, red) or calcein (green) to visualize mineralized bone matrix in relation to osteoblasts and osteoprogenitor populations. Images were acquired using a Zeiss LSM 880 confocal microscope at 10× magnification (scale bars = 100 µm).

***TPhP exposure induces structural abnormalities in vertebral body formation:*** Medaka exposed to 1.0 µM TPhP between 4–6 hpf and 14 dpf demonstrated gross skeletal changes including abnormal development of the axial skeleton centra, distorted formation of hemal and neural arches, and atypical formation of the caudal fin (Figure 1, Figure 2 and Figure 3). On average, the DMSO-treated fish had a significantly longer standard length of 6 mm (±0.18), while the TPhP-treated fish had a standard length of 5.18 mm (±0.07) (Supplemental Figure S1). While no gross changes to mineralization density were observed within the axial skeleton, there were gross structural differences between the TPhP- and DMSO-treated individuals. At 14 dpf, DMSO-treated individuals exhibited vertebral columns composed of evenly spaced, hourglass-shaped centra, with well-developed neural and hemal arches extending dorsally and ventrally from the rostral ends of each centrum. In comparison, the centra and neural and hemal arches of the TPhP-treated medaka exhibited reduced growth and irregular patterning of the axial centra resulting in enlargement of the IVL (Figure 1A,B). Quantitative morphological assessments of the centra demonstrate the severity of skeletal malformation throughout the axial skeleton of 14 dpf larvae. More specifically, measurements were performed on caudal centra 17–19 to quantify changes in area of mineralized centra and area of intervertebral ligament (IVL). On average, the DMSO centra had an area of 0.0118 (±0.00028) mm^2^, while the TPhP centra had a significantly smaller area of 0.0031 (±0.0039) mm^2^ (*p* ≥ 0.05). Reduction in caudal centra size following TPHP treatment likely resulted in an enlarged IVL, with DMSO-treated larvae exhibiting an IVL area of 0.0016 (±0.00026) mm^2^ and TPhP-treated larvae exhibiting a significantly larger IVL area of 0.0098 (±0.00041) mm^2^ (*p* ≥ 0.1) (Figure 1C,D).

***TPhP exposure results in morphological deficits in the caudal fin***: Delayed and/or abnormal development of the caudal fin was observed in TPhP-treated larvae in comparison to DMSO-treated controls (Figure 2A–C). Fins exhibited shorter lepidotrichia (rays) within the caudal fin and an obvious lack of mineralized centra within both the caudal and the caudal complex skeleton comprised of the preural centrum and urostyle [49]. TPhP-exposed individuals exhibited both attenuated and often lacked formation of both hemal and neural arch structures in caudal and the caudal complex. Similarly, the formation of the hypural structures that form the ventral to the caudal notochord and support extension of the caudal fin rays were attenuated compared to DMSO-treated medaka. On average, the DMSO hypural 1 (HP1) and hypural 2 (HP2) cartilaginous structures had areas of 0.0064 mm^2^ and 0.0118 mm^2^ (±0.0003 and 0.0005), respectively. The HP1 in the TPhP-treated fish was significantly smaller, with an average area of 0.0048 mm^2^ (±0.003), while the average area of the HP2 did not differ from the DMSO group. Furthermore, the parahypural (PH) structure in the TPhP treatment group was significantly shorter at 0.13 mm (±0.008) compared to the DMSO group at 0.15 mm (±0.004), and the hemal arch of PU2 (HPU2) was significantly shorter at 0.106 mm (±0.008) and had a smaller area of 0.003 mm^2^ (± 0.0003) compared to the HPU2 structure of the DMSO-treated fish: 0.143 mm (±0.009) and 0.004 mm^2^ (±0.0004), respectively (Figure 2C).

To further examine structural changes within the axial and caudal skeleton, we utilized select transgenic medaka lines to highlight alterations in bone cellularity of notochord development *tg*(*col2a:*nlGFP), formation of mineralized axial skeleton in relation to the composition of mesenchymal osteoprogenitors *tg*(*twist:*EGFP), immature osteoblasts *tg*(*col10a1*:nlGFP), and mature osteoblasts *tg*(*osx/sp7:*mCherry). Collagen type II is an extracellular matrix component of cartilaginous tissues, which forms a general scaffold for notochord development. In control animals, *Col2a1a:EGFP* (Figure 3A,B) expression is mainly observed in the notochord and cartilaginous components in the head. The expression is diffuse during the early stages of development but localizes to the nucleus pulposus during notochord differentiation when *col2a1* is shut off. With TPhP treatment, *col2a1*-EGFP + cells were dispersed and disorganized throughout the centra/vertebral column at 14 dpf. There was also a grossly observable increase in the size of the intervertebral ligaments (IVLs), predominately within centra comprising both the caudal and caudal complex. Notably, the hypural and parahypural cartilage was malformed but still exhibited EGFP expression within the hypertrophic chondrocytes. The *tg*(*twist:EGFP*) transgenic line (Figure 3C,D) marks sclerotome-derived mesenchymal stem cells (MSCs), which are densely concentrated within the intervertebral ligament (IVL) region. In DMSO-treated individuals, EGFP-positive cells were consistently observed within the defined boundary between the IVL and adjacent centra across all imaged segments. Following TPhP treatment, EGFP + cell expression was significantly attenuated throughout the axial skeleton. The remaining EGFP-positive cells were primarily concentrated at the IVL–centrum interface. A subset of these cells appeared to be infiltrating the mineralized centra, suggesting active migration and potential differentiation into osteoblasts. The *tg*(*col10a1:nlGFP*) line (Figure 1A,B and Figure 3E,F) expresses GFP in a heterogeneous population of osteoblast progenitors and pre-mature osteoblasts and in chondrocytes at later stages. In DMSO-treated individuals, nlGFP expression was observed in the neural and hemal arches, around the centra of vertebral bodies, and within the lepidotrichia of the caudal fin [38]. Imaging of TPhP-treated *tg*(*col10a1:nlGFP*) highlights the abnormal development of the axial skeleton and caudal fin. nlGFP + cells appeared more tightly packed together within the lepidotrichia and hypural cartilages of the caudal fin as well as in the axial centra. The centra were noticeably shorter, and the neural and hemal arches were deformed or not present at all. The *tg*(*osx:mCherry*) line (Figure 3G,H) labels premature and mature osteoblasts that mineralize bone matrix. In control individuals, there was strong expression in the neural and hemal arches of the centra and fin rays of the caudal fin. The same was observed in TPhP-treated individuals, although the mCherry expression highlighted irregular development of the arches in the anterior portion of the vertebral column and in many cases highlighted a lack of arches in the posterior portion, including the caudal and caudal complex centra. The lepidotrichia of the caudal fin developed with normal mCherry expression but with noticeably smaller and fewer lepidotrichia compared to the DMSO controls.

**Figure 4:**
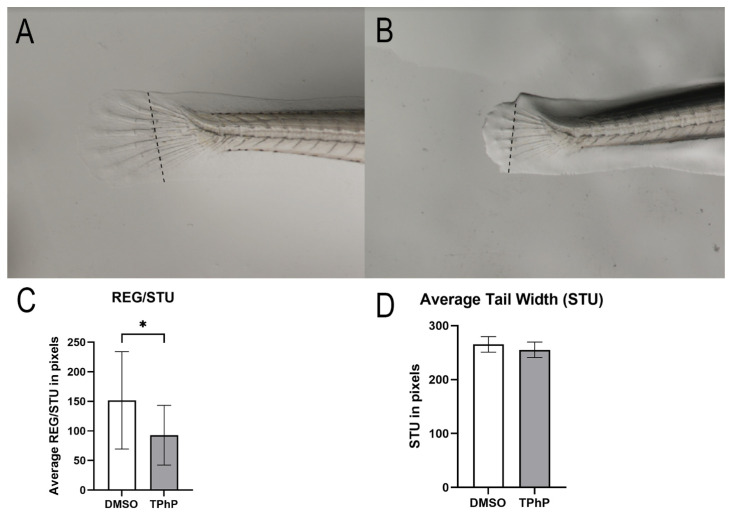
Morphological assessment of fin regrowth 5 dpa in whole-mount medaka larvae at 19 dpf. Representative individuals (*n* = 35–48 larvae per treatment) from DMSO- (**A**) and TPhP-exposed (**B**) groups are shown, 10× magnification. The REG/STU ratio is shown in (**C**); the STU measurement is shown in (**D**). The dotted line represents the STU measurement. Values represent the mean ± SD fold-change normalized to DMSO-treated controls. Asterisks indicate statistically significant differences (*p* < 0.05) using an unpaired, one-tailed Student’s *t*-test.

***TPhP exposure delays bone regeneration in the caudal fin:*** Fin regeneration studies were conducted to determine if TPhP exposure directly impacts axial growth. Medaka embryos were exposed to 1 µM TPhP as described above, and 14 dpf larvae were subjected to caudal fin clip. Larvae were subsequently grown in their respective conditions, in the presence of either DMSO or 1 µM TPhP in 1X ERM, and assessed 5 days post amputation (dpa) for fin regeneration. At 5 dpa, DMSO- and TPhP-exposed larvae were imaged, and the total regeneration area (REG) was measured. Following the protocol established by Cardeira et al. (2016) [48], we corrected for inter-specimen variation by also measuring the stump width (STU) for each individual. We then calculated the REG/STU ratio for each individual and averaged across each treatment group. The results indicate that TPhP exposure significantly (*p* < 0.05) attenuates regeneration with a REG/STU of 92.8 (±7.25) compared to the DMSO controls with a REG/STU ratio of 151.8 (±13.9) (Figure 4A), with no significant difference in total tail width (STU) of 265.3 (±14.39) for TPhP-treated larvae and 255.2 (±14.31) compared to the DMSO-treated controls (Figure 4B).

**Figure 5:**
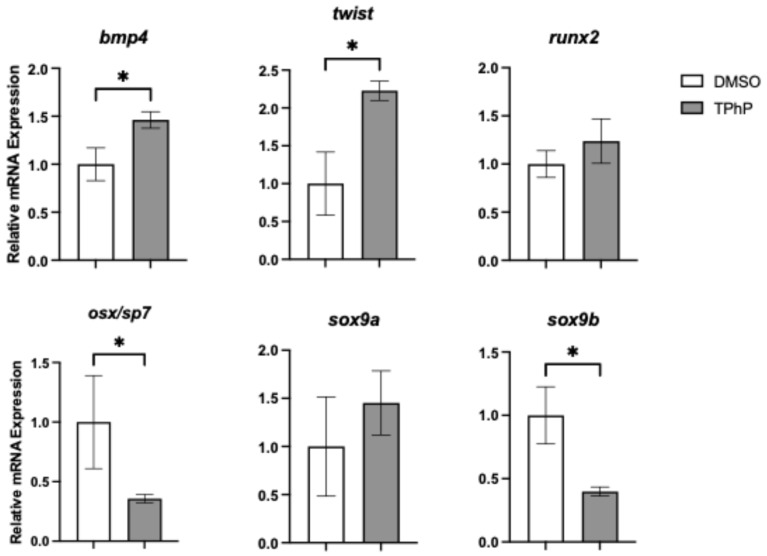
qPCR analysis of selected osteogenic transcription factors from axial tissue of medaka at 14 dpf. Data represent *n* = 3–4 technical replicates from 3–4 biological replicates, each consisting of pooled tissue from 15–20 larvae. Values represent the mean ± SD fold-change normalized to DMSO-treated controls. Asterisks indicate statistically significant differences (*p* < 0.05) using an unpaired, one-tailed Student’s *t*-test.

**Figure 6:**
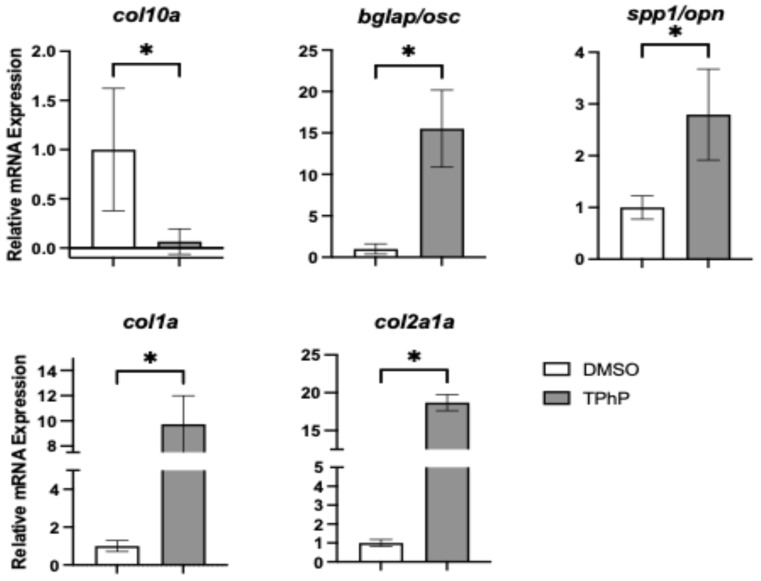
qPCR analysis of selected extracellular matrix (ECM) markers from axial tissue of medaka at 14 dpf. Each data point represents *n* = 3–4 technical replicates from 3–4 biological replicates, with tissue pooled from 15–20 larvae per replicate. Values represent the mean ± SD fold-change normalized to DMSO-treated controls. Asterisks indicate statistically significant differences (*p* < 0.05) using an unpaired, one-tailed Student’s *t*-test.

***TPhP exposure significantly impacts transcriptional regulators of osteogenesis and their downstream targets*:** To link observed deficits in bone mineralization to molecular changes, qPCR was performed on isolated axial tissues from DMSO- and TPhP-treated medaka to assess expression of key genes within the osteoblast gene regulatory network (Figure 5). Expression of *runx2*, a key early transcription factor in osteoblast differentiation, was not significantly altered by TPhP exposure. However, *osx/sp7*, which acts downstream of *twist1* and *runx2*, exhibited a significant 2.8-fold reduction in expression in TPhP-treated larvae compared to DMSO controls. Similarly, *sox9b*, a teleost co-ortholog of *sox9* essential for both osteoblast and chondrocyte differentiation, was significantly downregulated by 2.5-fold. To further investigate terminal osteoblast differentiation, expression of genes associated with the extracellular matrix formation and deposition were assessed (Figure 6). *Col10a1*, a marker of both osteoblasts and hypertrophic chondrocytes in teleosts, was significantly downregulated by 15.4-fold in TPhP-treated medaka relative to controls, consistent with observed attenuation in the expression of *col2a1a* in the transgenic reporter line. By comparison, all other extracellular markers were significantly upregulated in TPhP-treated larvae compared to DMSO controls. *bglap* expression was increased 15.5-fold, *spp1/opn* expression was increased 2.8-fold, *col1a* expression was increased 9.7-fold, and *col2a1a* expression was increased 18.7-fold.

***TPhP alters global osteochondral disease pathways:*** Using global transcriptional analysis, we demonstrated that exposure to 1 µM TPhP from early embryogenesis (4–6 hpf, blastula) through early larval development at 14 dpf (1st fry stage) [39] results in broad transcriptional alterations within the axial skeletal tissues. Bulk RNA-Seq analysis conducted with RNA isolated from the axial body of DMSO and 1 µM TPhP exposed medaka resulted in 2499 differentially regulated genes (>1.5-fold up or down), with 1428 transcripts with significantly up- and 1071 transcripts with significantly downregulated genes (q < 0.05, all RNA-Seq data have been deposited in GEO# GSE303479). Analysis of RNA-Seq data revealed modulation in expression of the number of osteochondral-related genes. Within the dataset, we observed induction of hyaluronan synthase (*has1*), *mir24a−2*, and osteoprotegerin (*opg*). Similarly, several downstream extracellular matrix (ECM) genes involved in bone and cartilage formation, including *col1a1*, *col2a1*, and *col11a*, were significantly upregulated. In line with qPCR results, TPhP treatment also led to downregulation of key transcriptional regulators such as *sox9*, *twist2*, and *sp7.* Notably, some of the most significantly downregulated genes in the TPhP-treated dataset were components of the mitochondrial electron transport chain, including *cytochrome c oxidase subunits I and II*; *NADH dehydrogenase subunits 1*, *2*, *and 3*; and *ATP synthase F0 subunits 6 and 8* (Supplemental Table S2).

**Figure 7:**
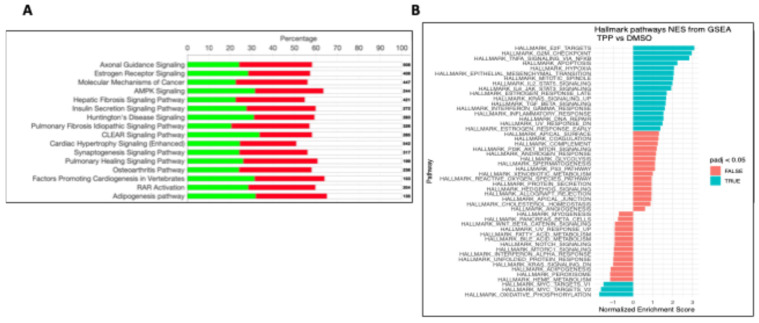
Bulk RNA-Seq gene analysis. (**A**) Ingenuity analysis, top canonical pathways, green upregulated genes, and red downregulated genes within each pathway; bold numbers represent total number of genes within the pathway. (**B**) Gene Set Enrichment Analysis TPhP vs. DMSO. Blue bars represent statistically significant positive enrichments at padj < 0.05; red bars represent pathway enrichments padj > 0.05.

Ingenuity Pathway Analysis (IPA) of the global RNA-Seq dataset demonstrated enrichment of select pathways associated with osteochrondral signaling. Specifically, estrogen receptor signaling osteoarthritis pathway, RAR signaling, and adipogenesis were among the top enriched canonical pathways, with 234/408, 137/236, 122/204, and 88/135 molecules (respectively) expressed within each pathway (Figure 7A, Supplemental Figure S2). Within disease and functions, “embryonic development/development of the body trunk” and organismal injury/death were among the top ten significantly activated categories, with z scores of 2.98 (199 molecules) and 3.99 (410 molecules) associated with each category, respectively IPA analysis demonstrated a significant enrichment of mitochondrial dysfunction with predicted disruption of cytochrome complex functionality and loss of ATP synthesis promoting mitochondrial myopathy and cytopathy (Supplemental Figure S3, Supplemental Table S2). Further, gene set enrichment assessments (GSEA) predicted enrichment (normalized enrichment value of −3.93) of a network describing modulation (downregulation) of oxidative phosphorylation. among other up- and downregulated pathways, including hallmarks of cell signaling, apoptosis, and inflammation (Figure 7B).

**Figure 8:**
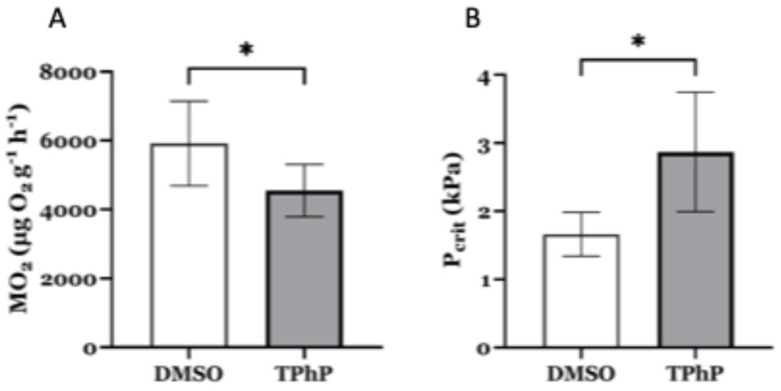
Metabolic function test in medaka larvae at 14 dpf. (**A**) Metabolic rate of DMSO- and TPhP-treated fish. (**B**) Critical oxygen tension (P_crit_) of DMSO and TPhP fish. Values represent the mean ± SD fold-change *n* = 5–8 larvae per treatment normalized to DMSO-treated controls. Asterisks indicate statistically significant differences (*p* < 0.05) using an unpaired, one-tailed Student’s *t*-test.

***Microrespirometry*:** Due to the dysregulation of a cluster of mitochondrial genes observed in bulk RNA-Seq experiments, we conducted whole-organism microrespirometry with DMSO- and 1 µM TPhP-treated medaka larvae at 14 dpf. We observed a significant shift in respirometry measures between the control and treatment groups. Specifically, the metabolic rate (MO_2_) was significantly lower in medaka hatchlings exposed to TPhP (Figure 8A) (*p* ≤ 0.05). In DMSO-treated fish, the average MO_2_ was 5919 (±549) μg O_2_ g^−1^ h^−1^. In TPhP-treated fish, the average MO_2_ was 4551 (±288) μg O_2_ g^−1^ h^−1^. Additionally, the P_crit_, the point where an animal can no longer maintain its normal oxygen consumption rate, was significantly increased in TPhP-treated larvae (*p* = 0.05). In DMSO-treated fish, the average P_crit_ was 1.6 ± 0.1 kPa, whereas the average P_crit_ in TPhP-treated fish was 2.8 ± 0.3 kPa (Figure 8B).

## 4. Discussion

Exposure to polybrominated diphenyl ether (PBDE) flame retardants has previously been associated with the suppression of endochondral ossification [35]. With the phase-out of these BFRs, it is important that we understand the skeletal toxicity of organophosphate ester (OPE) replacements. In the present study, we demonstrate that embryonic exposure to 1 uM of TPhP disrupts axial skeleton development and metabolism in medaka (*Oryzias latipes*). TPhP exposure resulted in deformed centra throughout the axial body and had a pronounced effect on centra formation/mineralization within the caudal fin and caudal complex and formation of the hypural structures supporting fin lepidotrichia. Gene expression analyses support modification of osteogenesis and highlight putative disruptions in mitochondrial function. Supporting these observations, we demonstrate that fin regeneration is significantly compromised in TPhP-exposed medaka larvae in conjunction with a demonstrated decreased metabolic performance, suggesting that TPhP exposures may promote energetic deficiencies.

Our findings indicate that TPhP exposure attenuates growth of the skeletal structures, including centra, neural, and hemal arches, and lepidotrichia, as evidenced by visualization of whole-mount Alizarin red and Alcian Blue staining and quantitative morphometry of skeletal structures. In marine medaka, developmental exposure to TPhP was observed to induced abnormal pectoral fin development and spinal curvature [36]. Similarly, zebrafish embryos exposed to TPhP previously demonstrated morphological defects including abnormal tail development and spinal deformities including lordosis, scoliosis, and kyphosis, shortened tail, and tail-tip damage [37]. Findings from this study further extend morphological defects within the axial skeleton of TPhP-treated medaka in addition to definitive defects throughout the axial vertebral column, resulting in poorly formed and mineralized caudal and caudal complex centra.

In teleosts, notochordal cells guide the initial mineralization and segmentation of the chordacentra along the vertebral column [50]. Confocal imaging of transgenic medaka used in this study reveals segmented patterns of positive ALC and calcein staining within the centra, suggesting that early mineralization of the notochordal sheath remains largely unaffected by TPhP exposure. Since chordal ossification occurs early, we propose that TPhP primarily interferes with ossification events regulating the lateral expansion of vertebral bodies along the rostral and caudal axes. At 14 dpf in *tg*(*twist:EGFP*) fish, sclerotome-derived mesenchymal stem cells (MSCs) were more widely dispersed across a larger intervertebral ligament (IVL) area compared to controls, with EGFP + cells extending beyond the IVL into the centra. Prior studies indicate these *tg*(*twist:EGFP*) cells are likely differentiating into perichordal osteoblasts [51]. Despite an apparent decrease in *tg*(*twist:EGFP*) MSCs in TPhP-treated fish, *twist* mRNA levels were significantly elevated relative to DMSO controls. Additionally, perichordal ossification along the neural and hemal arches showed diminished nlGFP + signal in *tg*(*col10a1:nlGFP*) fish and reduced mCherry + cell populations in *tg*(*osx/sp7:mCherry*) fish after TPhP exposure. These cellular findings align with molecular data demonstrating downregulation of osx/sp7 and col10a1 expression in treated medaka. Collectively, these observations suggest that TPhP disrupts intermediate to late stages of osteoblast differentiation and may also inhibit the maturation of osteoprogenitors into osteoblasts, offering a potential mechanism for TPhP-induced vertebral body malformations. Interestingly, our findings are consistent with fetal exposures to a formulated BFR mixture (combination of PBDE and HBCD mixtures) in Sprague–Dawley rats [52]. Offspring of dams fed various concentrations of this mixture in their diet developed several skeletal abnormalities, including offset ossification of sternebrae, unossified sternebrae and vertebrae, and incompletely fused ossified centrums. Furthermore, TPhP exposure detrimentally impacted endochondral differentiation in ex vivo murine limb buds more than BDE-47, a PBDE FR, and significantly decreasing cartilage condensation at concentrations as low as 1 μM [35]. At 3 μM, TPhP decreased longitudinal growth of the limb buds, limited the extent of cartilage template development, and inhibited the differentiation of hypertrophic chondrocytes and osteoblasts. Altogether, these indicate replacement OPFRs may have similar modes of toxicity as their brominated predecessors. 

Many teleosts can regenerate damaged fin tissue, including the medaka. Fish fins are structures that contain no musculature and are mostly made up of bony fin rays covered with thin epidermal cells. Individual fin rays consist of a pair of concave hemirays (lepidotrichium), which are then further composed of segments joined by ligaments, and the remaining spaces are filled with mesenchymal cells. Further elongation of fins are made by sequential addition of new segments to the distal end of each fin ray [53,54]. Adult fish can fully regenerate their missing bony structures 5–14 days after amputation (dpa). Fin regeneration is known to be impacted by contaminant exposure; for example, select heavy metals have been demonstrated to inhibit fin regeneration in killifish (*Fundulus confluentus*) and mullet (*Mugil cephalus*) [55,56]. Our study assessed the impact of TPhP exposure on fin regeneration in developing medaka larvae rather than adults. Wound healing occurs through the same mechanisms but on a shorter timeline, similar to the three days in zebrafish larvae [57], allowing us to explore a variation of skeletal development. Our results demonstrated that TPhP attenuated fin regeneration in medaka larvae, consistent with our observations of attenuated skeletal development.

To further explore the phenotypic changes observed in TPhP-treated medaka larvae, targeted qPCR analysis was performed on axial tissue at 14 dpf, focusing on key genes within the osteogenic regulatory network. Exposure to TPhP significantly reduced the expression of osx/sp7, sox9b, and col10a. Both osx/sp7 and sox9b serve as early markers of osteogenic differentiation, and the skeletal abnormalities observed in the centra of TPhP-treated larvae closely resemble those observed in medaka with osx/sp7 knockdown via morpholinos. Specifically, osx/sp7 morpholino-treated fish show diminished formation of mineralized centra [51]. Sox9 is a critical transcription factor for chondrocyte differentiation and skeletogenesis. Unlike many vertebrates, teleost fish possess two co-orthologs, sox9a and sox9b, which have distinct roles in bone development. In zebrafish and related teleost species, sox9b is essential for the transition of epithelial cartilages into bone. Loss of sox9b disrupts this process and impairs the formation of skeletal elements such as cranial cartilage and the jaw [58,59]. Dong et al. [60] observed attenuated medaka hypural development in tandem with significantly decreased *sox9a* (equivalent to the zebrafish *sox9b* ortholog) expression in response to TCDD exposure. Additionally, *sox9b* zebrafish mutants exhibited malformed pharyngeal cartilage organs, indicating that *sox9b* is necessary for these cartilages to acquire their normal cell number either through cellular apoptosis or proliferation [61]. During early development in medaka, *col10a1* is expressed in chondrocytes and pre-osteoblasts. Tan et al. [62] developed a *col10a1* medaka mutant with a 5 bp deletion, of which the heterozygous mutants exhibited reduced *col10a1* expression compared to the wild types and exhibited shorter vertebral bodies and body length, similar to our observations from TPhP exposure. In ex vivo murine limb bud cultures, TPhP limited chondrogenesis and inhibited osteogenesis and suppressed the expression of *sox9* and *sp7* [35]. Interestingly, the expression of *twist* and *bmp4* was induced following TPhP exposure. Both genes serve as osteogenic drivers promoting differentiation of mesenchymal stem cells towards an osteoblast lineage [63]. In conjunction, we additionally observed a significant increase in the expression of markers for terminally differentiated osteoblast and formation of extracellular matrix, including *spp1*, *bglap*, *col2a1*, and *col1a*. Indeed, induction of these genes is counterintuitive to a reduction in centra formation and loss of mineralized bone structures within the axial skeleton. By comparison, exposure of medaka embryos/larvae to TCDD results in attenuation of terminal differentiation and extracellular matrix markers following developmental exposure consistent with loss of skeletal mineralization [38]. With TPhP exposures, upregulation of select transcriptional regulators (*twist* and *bmp4*) and extracellular matrix production may indicate a compensatory response to compromised osteogenesis and centra formation and mineralization. Alternatively, *osx*, *sox9b*, and *col10a* each have essential roles in chondrogenesis and osteochondral disease including osteoarthritis, where over-expression of *col10* has been noted [64]. For example, *osx*, while well-known for its role in osteogenesis, is demonstrated to facilitate coupling of terminal cartilage differentiation and endochondral ossification in addition to ossification and degradation of cartilage matrix [65]. Similarly, *sox9* serves as an essential transcriptional regulator of chondrogenesis in conjunction with expression of *col10*, and both are implicated with osteochondral pathogenic phenotypes including osteoarthritis [66,67].

In addition to targeted gene expression, we conducted bulk RNA-Seq on DMSO/TPhP-treated axial skeletal tissue. Supporting our targeted approach, we observed enrichment of select osteochondral pathways including osteoarthritis. The osteoarthritis pathway appears to be driven through increased expression of select osteogenic transcription factors including *osx*, *cepb-β*, and *dlx5* in conjunction with dysregulation of several upstream cell signaling modulators associated with osteogenesis including *mantn*, *grem1*, *bmp*, *FGF*, *Apelin*, and *wnt* (Supplemental Figure S2). Interestingly, both IPA and GSEA demonstrate disruptions of mitochondrial function and oxidative phosphorylation with an end result of decreased energy production. This observation is consistent with previous reports that TPhP and other flame retardants may impact mitochondrial function [68].

To further investigate how loss of mitochondrial function may impact whole-organismal physiology, we subsequently conducted whole larval respirometry. The joint observation of decreased metabolic rate and increased P_crit_ suggest that the TPhP-treated fish are more energetically compromised compared to the control (DMSO) fish. Previous studies report that exposure to endocrine disrupting OPFRs, such as TPhP, may predispose individuals to metabolic disorders. Le et al. [69] demonstrated that mouse liver cells exposed to aryl- and chlorinated-OPFRs developed impaired mitochondrial structure, increased ROS production, decreased mitochondrial membrane potential (MMP), inhibition of the electron transport chain (ETC), and significantly decreased mitochondrial ATP production rate. Chinese hamster ovary (CHO-k1) cells exposed to aryl- and alkyl-OPFRs also demonstrated mitochondrial impairment as well as cell death through different mechanisms [70]. In the Japanese quail, there have been observations of TPhP significantly reducing metabolic rate and growth in chicks [71]. Decreased growth rate was also observed in weanling rats fed 1% ww of TPhP [72]. Considering the link between metabolic rate and skeletal growth, our findings further suggest that early-life exposure to TPhP disrupts this balance, likely by impairing mitochondrial function. Thus, exposure to TPhP and other OPFRs may increase the risk of bone metabolic disorders (BMD) such as osteoporosis, osteomalacia, osteogenesis imperfecta, metabolic arthritis, and more.

## 5. Conclusions

Together, our findings provide compelling evidence that TPhP disrupts key regulatory processes involved in skeletal development, particularly by impairing osteoblast differentiation and vertebral body formation during early-life stages. The observed molecular and cellular disruptions, including altered expression of osteogenic markers such as *osx/sp7*, *col10a1*, and *twist*, underscore the sensitivity of the developing skeleton to environmental toxicants. Importantly, the use of transgenic medaka models enabled detailed visualization of these perturbations in vivo, offering mechanistic insight into how TPhP interferes with conserved pathways of vertebrate skeletogenesis. Given the widespread presence of organophosphate flame retardants in the environment, our data raise significant concerns about their safety. These findings not only support the use of teleost models for toxicological assessment but also emphasize the urgent need for regulatory frameworks that consider the long-term skeletal risks associated with emerging replacement chemicals.

## Data Availability

The data presented in this study are available on request from the corresponding author. Bulk RNA-Seq data are available through GEO, accession #GSE303479.

## Supplementary Materials

The following supporting information can be downloaded at https://www.mdpi.com/article/10.3390/toxics13080654/s1. Table S1: qPCR Primer sequences; Table S2: Down regulation of mitochondrial genes following TPhP treatment; Figure S1: Larval growth; Figure S2: IPA osteoarthritis pathway TPhP vs. DMSO. Figure S3: Graphical output of IPA Mitochondrial disruption of TPhP vs. DMSO bulk RNASeq datasets.
