# Supplementary material for: Embryonic Exposure to TPhP Elicits Osteotoxicity via Metabolic Disruption in Oryzias latipes
Source: Toxics. 2025 Jul 31;13(8):654. doi: 10.3390/toxics13080654 (PMC12390264; doi:10.3390/toxics13080654)

Supplemental Table S1. qPCR Primer sequences

Gene	Primer Sequence (5' – 3')	Transcript ID	Primer efficiency
bmp4	CAACGATGATAGCCCCCTAA (forward)	ENSORLG00000013304	100.00%
	GCTGGTTGTGTGGAGTCTGA (reverse)		
twist 1	AAAACGGGGAGGACTCAGAT (forward)	ENSORLG00000007251	98.90%
	GAGGGCAAAGTGGGGATAAT (reverse)		
runx2	GAGCTTCACGCTGACAATCA (forward)	ENSORLG00000010169	101.80%
	CTCTCACTCGCATCCTTTCC (reverse)		
osx/sp7	GCCTCTGACCTTCAAACAGC (forward)	ENSORLG00000005215	100.00%
	GGCTTTGGACACGAGAAGAG (reverse)		
sox9α	CTCAACATCGACTTCCGTGA (forward)	ENSORLG00000007960	102.10%
	GTCGAACGTCTCAATGTGGG (reverse)		
sox9β	AGCTACGGGTCCTTCAACCT (forward)	Gene ID: 100125804	98.60%
	GCTGTAGTAAGAGTTGGCAC (reverse)		
col10α	TGCTCCAGGTATTCCAGGTC (forward)	ENSORLG00000013436	95.80%
	GTTTGGCTTTTGGAACCTGA (reverse)		
bglap/osc	AAAGAGACCTGGCTGCTGTT (forward)	ENSORLG00000025538	105.50%
	GCCCTCAGTGTCAGCCATTT (reverse)		
spp1/opn	TGTCCTCCAGCGTATCTCAA (forward)	ENSORLG00000020900	102.00%
	CCTCCTCCTCTTCCTCCTGT (reverse)		
col1α	GGTTCTGCTGGTAACGATGG (forward)	ENSORLG00000017013	97.4
	CCAGGCATTCCAATAAGACC (reverse)		
col2a1	ACAGCCGCTTCACCTACAGT (forward)	ENSORLG00000012738	99.60%
	GGGCCTATGTCAACTCCAAA (reverse)		
rpl13a	CTATGACCAATAGGAAGAGCAACC (froward)	ENSORLG00000014599	103.00%
	GCAGAGTATATGACCAGGTGGAA (reverse)		

[a] Primers used for qPCR analysis. Gene names, Ensembl and/or NCBI GenBank Accession IDs, and primer efficiencies are included for each gene examined.

[b] Coding sequences were obtained from the Ensembl (ID= ENSORLGXXXXXXXXXXX) and/or NCBI GenBank databases (ID= NM/XM_XXXXXXXX.X). Forward and reverse primer were confirmed by Blast analysis against the NCBI database and/or Ensembl medaka genome. Japanese medaka HdrR in Ensembl release 114, May 2025.

[c] Primer efficiencies were conducted for each gene-specific primer using 10-fold cDNA dilutions. Cycle threshold (C_t) values were plotted against log(cDNA) concentrations, and "Percent Efficiency" was calculated using the following equation,

$$\%\ \text{Efficiency} = [[(\text{Dilution factor})^{(\frac{1}{-\text{slope}})}] - 1] \times 100$$

where the dilution factor is 10, and the slope is based on a linear regression trendline.

All sequences based on Japanese medaka HdrR in Ensembl release 114, May 2025

Supplemental Table S2. Down regulation of mitochondrial genes following TPhP treatment.

Gene Symbol	Expr Log 2 Ration	Entrez name	Transcript ID
cox2	-8.012550544	Cytochrome c oxidase subunit II	ENSORLG00000021766
nd2	-8.012449194	NADH dehydrogenase subunit 2	ENSORLG00000021757
nd1	-7.689928322	NADH dehydrogenase subunit 1	ENSORLG00000021753
atp6	-7.219403361	ATP synthase F0 subunit 6	ENSORLG00000021769
nd3	-6.27968668	NADH dehydrogenase subunit 3	ENSORLG00000021772
atp8	-4.151807105	ATP synthase F0 subunit 8	ENSORLG00000021768
nr1d4a	-2.861679239	Nuclear receptor subfamily 1 group D member 1	ENSORLG00000007837
cox1	-2.487641734	Cytochrome c oxidase subunit I	ENSORLG00000021763
nr1d4b	-1.990600945	Nuclear receptor subfamily 1 group D member 1	ENSORLG00000015399

[a] Transcript ID obtained from the Ensembl (ID= ENSORLGXXXXXXXXXXX), Japanese medaka HdrR in Ensembl release 114, May 2025

SUPPLEMENTAL FIGURES.

Supplemental Figure S1. Larval growth. Stunted growth observed in TPhP-treated medaka at 20 dpf. Values represent the mean ± SD fold change n = 8-10 larvae per treatment. Asterisks indicate statistically significant differences ($P < 0.05$) using an unpaired, one-tailed Student's t-test

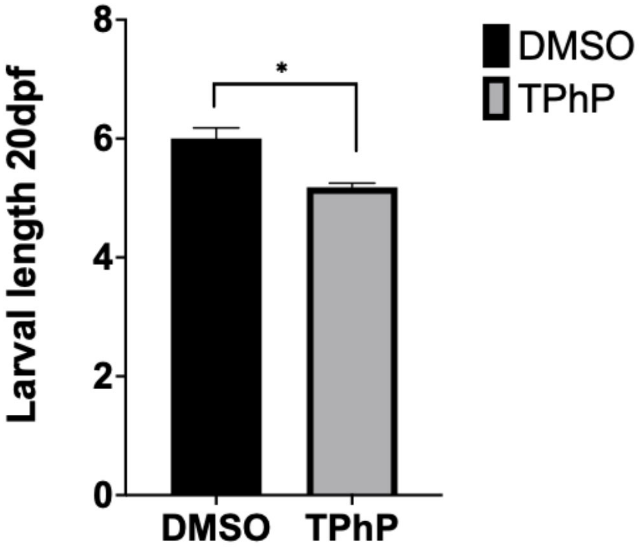

Supplemental Figure S2. IPA osteoarthritis pathway TPhP vs DMSO. Within IPA analysis, TPhP exposures result in enrichment of the osteoarthritis pathway in comparison to DMSO treatments. Over all attenuation in osteochondral signaling is predicted with 137/236 genes differentially expressed with in the defined IPA pathway resulting in a Z score of 2.45. Color key indicates degree of up/down regulation for differentially expressed genes within the bulk RNASeq dataset and predicted activation/inhibition of associated gene and functions.

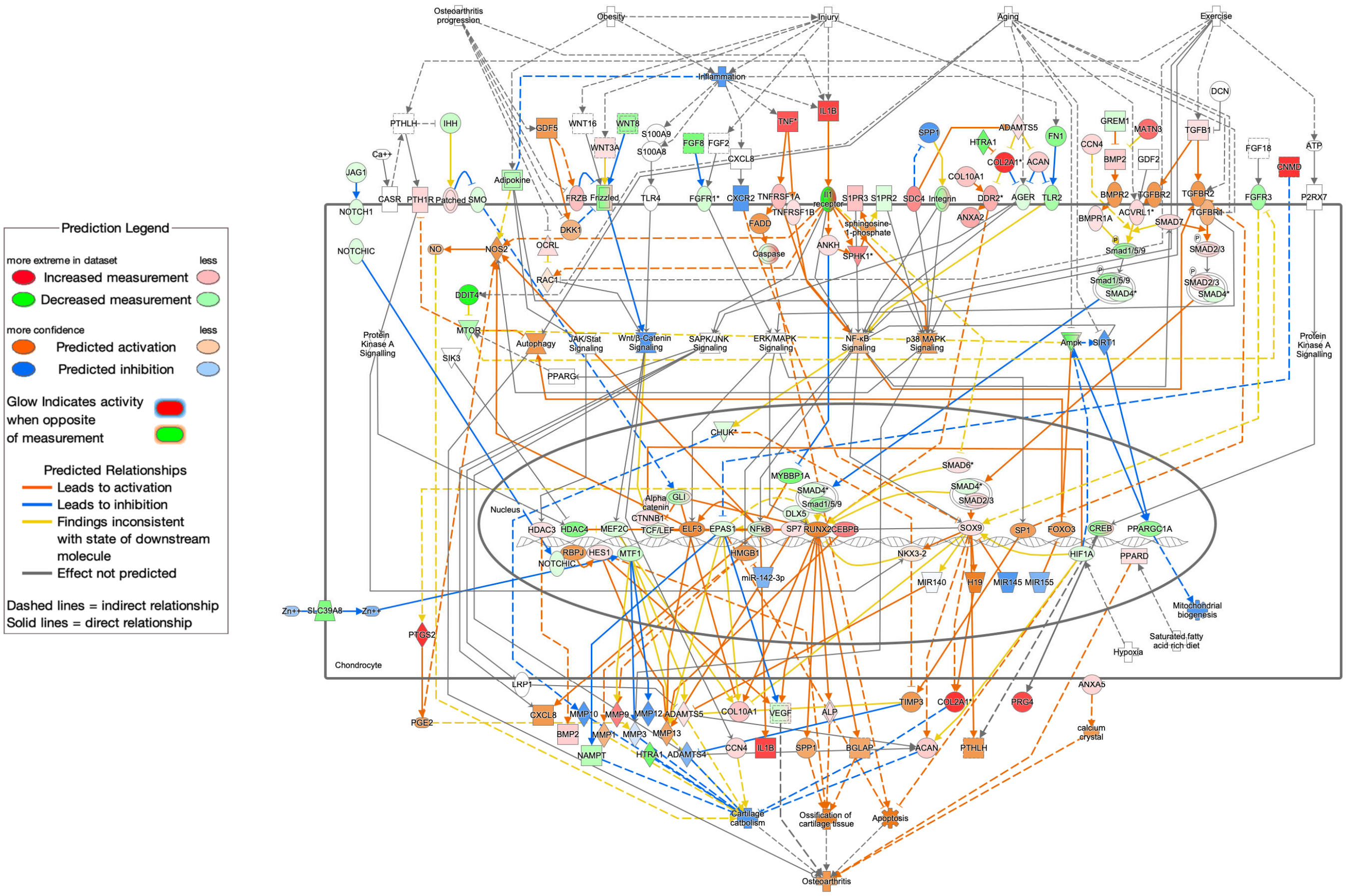

Supplemental Figure S3. Graphical output of IPA Mitochondrial disruption of TPhP vs DMSO bulk RNASeq datasets. Green represents overall down regulation of mitochondrial function and/or individual mitochondrial genes within each complex of the electron transport chain. Red represents up regulation of select mitochondrial genes. Blue represents predicted down regulation of select genes. Mixed red and green suggest both up and down regulated gene set with in each mitochondrial complex.

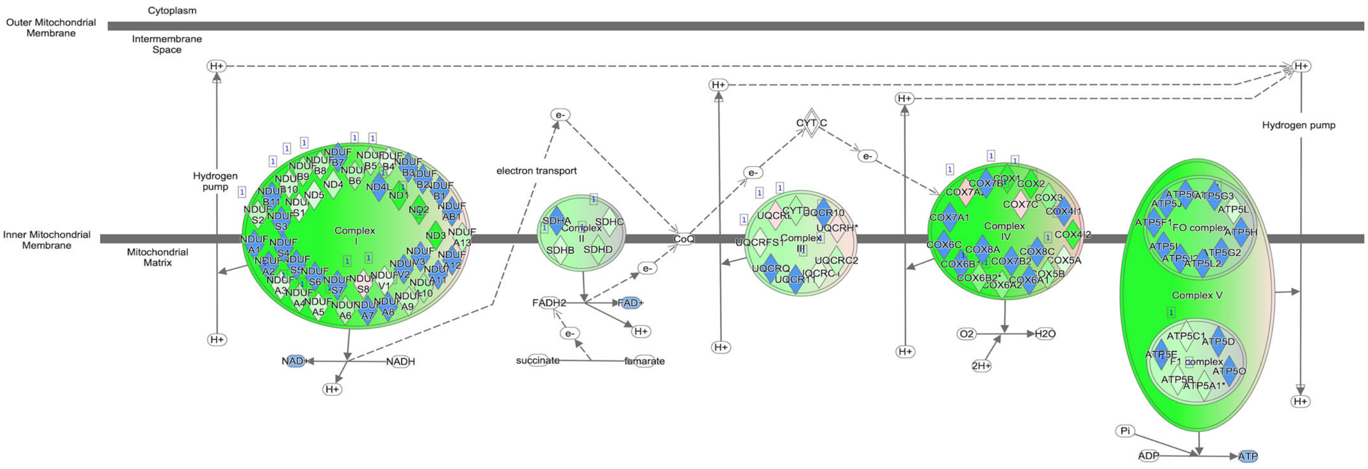

Supplement: Supplementary file 1 [file toxics-13-00654-s001.zip › toxics-3728977-supplementary.pdf]
